# Clinical Outcome in Pediatric Patients with Philadelphia Chromosome Positive ALL Treated with Tyrosine Kinase Inhibitors Plus Chemotherapy—The Experience of a Polish Pediatric Leukemia and Lymphoma Study Group

**DOI:** 10.3390/cancers12123751

**Published:** 2020-12-13

**Authors:** Joanna Zawitkowska, Monika Lejman, Marcin Płonowski, Joanna Bulsa, Tomasz Szczepański, Michał Romiszewski, Agnieszka Mizia-Malarz, Katarzyna Derwich, Grażyna Karolczyk, Tomasz Ociepa, Magdalena Ćwiklińska, Joanna Trelińska, Joanna Owoc-Lempach, Ninela Irga-Jaworska, Anna Małecka, Katarzyna Machnik, Justyna Urbańska-Rakus, Radosław Chaber, Jerzy Kowalczyk, Wojciech Młynarski

**Affiliations:** 1 Department of Pediatric Hematology, Oncology and Transplantology, Medical University of Lublin, 20-059 Lublin, Poland; jerzy.kowalczyk@uszd.lublin.pl; 2 Laboratory of Genetic Diagnostics, Medical University of Lublin, 20-059 Lublin, Poland; monika.lejman@umlub.pl; 3 Department of Pediatric Oncology, Hematology, Medical University of Bialystok, 15-089 Bialystok, Poland; marcin.plonowski@udsk.pl; 4 Department of Pediatrics, Hematology and Oncology, Medical University of Silesia, 40-752 Katowice, Poland; jbulsa@szpital.zabrze.pl (J.B.); szczep57@poczta.onet.pl (T.S.); 5 Department of Hematology and Pediatrics, Medical University of Warsaw, 02-091 Warsaw, Poland; michal.romiszewski@uckwum.pl; 6 Department of Pediatric Oncology, Hematology and Chemotherapy, Medical University of Silesia, 40-752 Katowice, Poland; amizia-malarz@sum.edu.pl; 7 Department of Pediatric Oncology, Hematology and Transplantology, Poznan University of Medical Sciences, 61-701 Poznan, Poland; kderwich@ump.edu.pl; 8 Department of Pediatric Oncology and Hematology, School of Medicine Jan Kochanowski University, 25-317 Kielce, Poland; grazyna.karolczyk@wszzkielce.pl; 9 Department of Pediatrics, Hematology and Oncology, Pomeranian Medical University of Szczecin, 70-204 Szczecin, Poland; tociepa@sci.pum.edu.pl; 10 Department of Pediatric Oncology and Hematology, Jagiellonian University Medical College, 31-008 Krakow, Poland; mcwiklinska@usdk.pl; 11 Department of Pediatrics, Oncology & Hematology, Medical University of Lodz, 90-647 Lodz, Poland; joanna.trelinska@umed.lodz.pl (J.T.); wojciech.mlynarski@umed.lodz.pl (W.M.); 12 Department of Pediatric Transplantology, Oncology, Hematology, Wroclaw Medical University, 50-367 Wroclaw, Poland; joanna.owoc-lempach@umed.wroc.pl; 13 Department of Pediatrics, Hematology, Oncology and Endocrinology, Medical University of Gdansk, 80-210 Gdansk, Poland; nirga@gumed.edu.pl (N.I.-J.); a.malecka@gumed.edu.pl (A.M.); 14 John Paul II Upper Silesian Child Health Centre, Department of Pediatric Hematology and Oncology, Medical University of Silesia, 40-752 Katowice, Poland; k.machnik@zsm.com.pl (K.M.); hematologia.dz@zsm.com.pl (J.U.-R.); 15 Clinic of Pediatric Oncology and Hematology, Faculty of Medicine, University of Rzeszow, 35-959 Rzeszow, Poland; rchaber@ur.edu.pl

**Keywords:** ALL Ph+, children, tyrosine kinase inhibitors (TKIs), toxicity, outcome

## Abstract

**Simple Summary:**

Philadelphia chromosome positive acute lymphoblastic leukemia (ALL Ph+) is rare in children, but outcomes are still poor. The aim of our study was to analyze the toxicity events and results of children with ALL Ph+ treated according to the EsPhALL2010 protocol (the European intergroup study of post induction treatment of Philadelphia chromosome positive ALL) in Poland between the years 2012 and 2019. Our treatment outcomes are still disappointing compared to those in other reports. Improvements in supportive care and emphasis placed on the determination of MRD at successive time points, which will impact decisions on therapy, may be required.

**Abstract:**

The treatment of children with Philadelphia chromosome positive acute lymphoblastic leukemia (ALL Ph+) is currently unsuccessful. The use of tyrosine kinase inhibitors (TKIs) combined with chemotherapy has modernized ALL Ph+ therapy and appears to improve clinical outcome. We report herein the toxicity events and results of children with ALL Ph+ treated according to the EsPhALL2010 protocol (the European intergroup study of post-induction treatment of Philadelphia chromosome positive ALL) in 15 hemato-oncological centers in Poland between the years 2012 and 2019. The study group included 31 patients, aged 1–18 years, with newly diagnosed ALL Ph+. All patients received TKIs. Imatinib was used in 30 patients, and ponatinib was applied in one child due to T315I and M244V mutation. During therapy, imatinib was replaced with dasatinib in three children. The overall survival of children with ALL Ph+ treated according to the EsPhALL2010 protocol was 74.1% and event-free survival was 54.2% after five years. The cumulative death risk of the study group at five years was estimated at 25.9%, and its cumulative relapse risk was 30%. Our treatment outcomes are still disappointing compared to other reports. Improvements in supportive care and emphasis placed on the determination of minimal residual disease at successive time points, which will impact decisions on therapy, may be required.

## 1. Introduction

Philadelphia chromosome positive acute lymphoblastic leukemia (ALL Ph+) accounts for only 2–5% of the pediatric population with ALL, but is a major therapeutic problem for clinicians [1]. The treatment of children with ALL Ph+ is currently unsuccessful, despite multidrug chemotherapy and allogenic hematopoietic stem cell transplantation (HSCT). This is why these patients are classified as high risk. The use of tyrosine kinase inhibitors (TKIs) combined with chemotherapy has modernized the therapy of ALL Ph+ and appears to improve clinical outcome [2,3,4]. The literature indicate that children with ALL Ph+ treated with imatinib plus intensive chemotherapy followed by HSCT have better outcomes, especially in the poor-risk group. Some authors reported that the five years’ event-free survival (EFS) was 81.3% and overall survival (OS) was 87.5% in these patients, compared to 55% and 62%, respectively, in the pre-imatinib era [1,5]. Some authors have suggested that imatinib integrated with conventional chemotherapy might increase the severity of toxic events. Therefore, a balance should be found between preventing relapse and increasing the risk of serious complications [6]. Currently, the main goal of international studies is to determine the most appropriate use of chemotherapy, HSCT, and TKIs in children with ALL Ph+ [7,8].

In this study, we report the toxicity events and results of children with ALL Ph+ treated according to the EsPhALL2010 protocol (the European intergroup study of postinduction treatment of Philadelphia chromosome positive ALL) in Poland between the years 2012 and 2019.

## 2. Results

### 2.1. Patient Characteristics

The demographic data of the analyzed patients are summarized in Table 1. The study group was characterized by the following features: most children (58%) were male, the immunophenotype of the B-cell precursor line prevailed (93.5%), most subjects (87.1%) had no infiltration of the central nervous system (CNS) and most (71%) had an initial white blood cell (WBC) count of ≥20,000/µL. Cytogenetic analyses of bone marrow samples obtained at the time of diagnosis were performed using conventional methods. Initial FISH (fluorescent in situ hybridization) analysis for identification of *BCR/ABL1* (Breakpoint cluster region-Abelson murine leukemia viral oncogene homolog 1) fusion was performed and t(9; 22)(q34; q11.2) translocation was identified in all cases. None of the cases had translocation t(12; 21)(p13; q22) or t(1; 19)(q23; p13), or 11q23 rearrangements. Hyperdiploidy with >50 chromosomes was found in three cases. Hypodiploidy was observed in two cases. A complex karyotype (above three aberrations) was observed in 10 cases, and aberrations of chromosome 7 with loss of IKZF1 were observed in four cases. A somatic karyotype with t(9; 22)(q34; q11.2) was found in 22 cases.

About half of the children had a good response to prednisone on Day 8, but they had poor myelogram results (M2 or M3 marrow and MRD (minimal residual disease) > 10%) on Day 15. A total of 67.7% patients were stratified as the poor-risk group. Cranial radiotherapy was applied to four (12.9%) patients.

All patients received tyrosine kinase inhibitors (TKIs). Imatinib was used in 30 patients, and ponatinib was applied in one child due to T315I and M244V mutation. During therapy, imatinib was replaced with dasatinib in three children (in two patients, due to progression of disease into the CNS; in one patient, due to the escalation of the BCR/ABL1 transcript before the HR-3′ course). Imatinib was well tolerated. We mainly observed the following complications: infection (12 patients; 38.7%), hepatotoxicity (11 patients; 35.5%), and gastrotoxicity (seven patients; 22.6%). The patients who received dasatinib due to progression of the disease into the CNS had the following severe complications: dermatitis (one child), gastrotoxicity (two children), hepatotoxicity (two), infection (two), and neurologic events (one). The patient who required dasatinib because of transcript escalation had a fungal infection and liver toxicity. The child who received ponatinib presented severe toxic effects, such as dermatitis, hepatotoxicity, and gastrotoxicity. Table 2 presents the grade of the toxicity for each patient.

Stem cell transplantation was performed in 23 (74.2%) children—in five patients (16.1%) from a matched sibling donor (MSD) and in 18 patients (58.1%) from a matched unrelated donor (MUD).

### 2.2. Treatment Results

**Figure 1:**
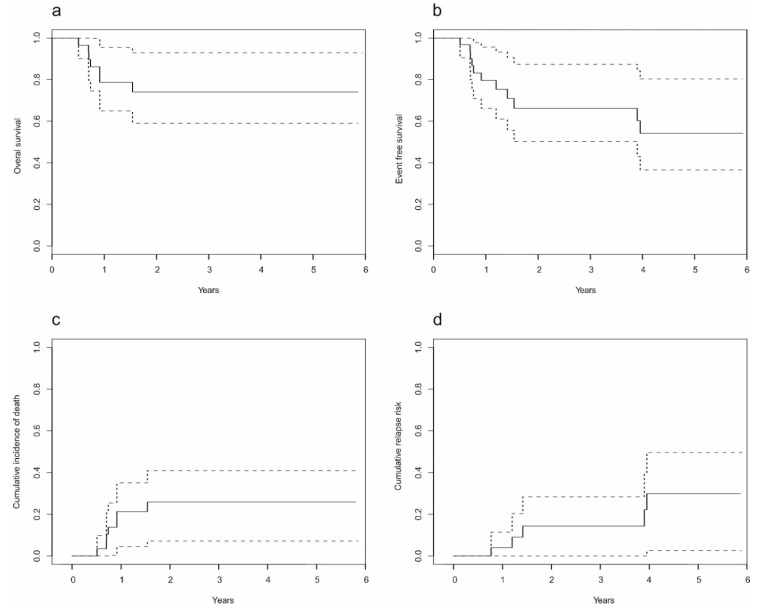
Analysis of the entire cohort of children with ALL Ph+: (**a**) overall survival; (**b**) event-free survival; (**c**) cumulative incidence of deaths; (**d**) cumulative incidence of relapse. Solid line in the figure represents cumulative incidence, while dotted lines represent upper and lower confidence interval (95%) for this cumulative incidence.

The median follow-up time for the entire group was 21.5 months. Relapse occurred in five children (one boy, four girls), and two of them were treated with MUD-SCT(matched unrelated donor stem cell transplantation). The site of recurrence was the CNS in one child, the testicles in one child, and bone marrow in three children. Seven out of the 31 (22.6%) children died. Four of them died due to complications after SCT, one child died due to progressive disease, one child died as a consequence of a septic shock, and one child died of heart failure. The overall survival (OS) of children with ALL Ph+ treated with the EsPhALL2010 protocol was 74.1% (SE (standard error) 0.086; 95% CI (confidence interval) 59.1–92.9) after five years in Poland (Figure 1a). The event-free survival (EFS) rate of the analyzed group of patients was 54.2% (SE 0.11; 95% CI 36.6–80.3) after five years (Figure 1b). The cumulative death risk of the study group at five years was estimated at 25.9% (SE 0.09; 95% CI 7.1–40.9), and cumulative relapse risk of the same was 30.0% (SE 0.118; 95% CI 2.5–49.7) (Figure 1c,d).

## 3. Discussion

Currently, the use of intensive chemotherapy and steroids cures most children with acute lymphoblastic leukemia. The development of genetic methods has led to the identification of molecular ALL subgroups, such as BCR/ABL1 chromosomal aberration, with pronounced biological and clinical features associated with poor results of treatment [9,10]. An optimal solution that would combine available methods, TKIs, chemotherapy, and SCT in children with ALL Ph+ is still being sought. Monitoring of MRD levels, evaluated by flow cytometry and/or PCR test, during and at the end of the induction phase of therapy is useful for assessing treatment response and making SCT decisions [11,12,13,14].

Our study is an analysis of the results and toxicity of Polish pediatric ALL Ph+ patients treated with tyrosine kinase inhibitors plus chemotherapy. The advantage of this study is that it concerns a rare subgroup of patients, and its results may be useful in clinical practice. There is a difference between the results from our cohort and those from other studies that may arise from the use of nonidentical methods to monitor MRD during the treatment. Another aspect was the time points at which the MRD determinations were made and used in patient stratification. In our study, MRD levels were only measured by a flow cytometer on Day 15 of the induction phase due to limited resources. In other studies, MRD was assessed by PCR at three time points. PCR-MRD is a more sensitive method, and additionally, assessing the MRD more often resulted in a more accurate assessment of treatment response and contributed to treatment decisions [15,16,17].

Biondi et al. published results from the EsPhALL2010 study, which was done on 11 study groups and included a large number of patients (*n* = 155) between the years 2010 and 2014. Molecular assessments of MRD (PCR-MRD) levels were made available only for some national groups and were analyzed at three time points: at the end of the induction phase, the end of the consolidation phase, and the end of high-risk block 3. The authors reported that the five-year overall survival was 71.8% (63.5–78.5) and five-year event-free survival was 57.0% (95% CI 48.5–64.6), and the cumulative relapse risk was 26.9% (19.8–34.4) after five years. The authors suggested that imatinib given at front-line induction and continued throughout chemotherapy might increase the severity of adverse events, and the most common complications were infection (39%), gastrotoxicity (6%), and osteonecrosis (5%). A total of 25/155 children (16%) died, and the cumulative death risk was 16.1% (95% CI 10.8–22.4). The authors reported that 52% had serious adverse events such as infection (8%), multiple-organ failure (1%), and SCT-related events (7%). Our analysis showed that imatinib was well tolerated, and the toxicity was mainly grade II. In our study, seven children died (22.6%), mainly related to SCT. The cumulative death risk was higher than that in Biondi’s study. We explain this as being due to the more accurate MRD measurement method used by Biondi and insufficient supportive therapy in Poland. The authors also compared the results of the EsPhALL2010 and EsPhALL2004 protocols. The differences were in prior and longer exposure to imatinib in all patients and in the limitation of eligibility for transplantation. Prior exposure to imatinib occurred from day 15 of induction as opposed to postinduction with EsPhALL2004. The potential total exposure to imatinib was 24 months for nontransplant patients and 15 months for transplant recipients, while in study EsPhALL2004, the total exposure was three or five months depending on whether patients had HSCT or not. The overall survival and the event-free rates were similar between these studies [6,17].

Imatinib has also found applications in patients with ABL-class fusions other than BCR-ABL1. Cario et al. analyzed 46 pediatric patients with Ph-like ALL treated according to AIEOP-BFM ALL (international collaborative treatment protocol for children and adolescents with acute lymphoblastic leukemia) 2000 and 2009 protocols. The stratification by risk group was the main difference between the two protocols. The high risk (HR) criteria were minimal residual disease levels (MRD) of ≥10^−3^ at Day 78 (MRD-HR), no complete remission (CR) at Day 33, and poor prednisone response (PPR). In AIEOP-BFM ALL 2009, patients were additionally assigned to the high-risk group (HR) when their PCR-MRD was >5 × 10^−4^ at Day 33 and was still measurable at Day 78 and/or if their FCM-MRD at day 15 day was >10%. For the entire group of 46 children, the five-year event-free survival was 49.1 ± 8.9% and overall survival was 69.6 ± 7.8%; the cumulative incidence of relapse was 25.6 ± 8.2%, and treatment-related mortality was 20.8 ± 6.8% [9].

Shen et al. published a study in which patients with ALL Ph+, aged 0 to 18 years, were randomized to receive chemotherapy combined with daily dasatinib at a dosage of 80 mg/m^2^ per day (*n* = 92) or imatinib at 300 mg/m^2^ per day (*n* = 97) from diagnosis to the end of therapy. The authors reported that therapy including dasatinib gave excellent results compared with imatinib, especially in patients with CNS infiltration. In this study, there were no significant differences in the incidence of severe complications between the two analyzed groups of patients [18]. Our study reported observations of serious side effects in children who received dasatinib. However, our observations are not conclusive as dasatinib was only used in three children. One of those patients died due to toxicity after SCT. McCafferty et al. reviewed the efficacy and tolerability of dasatinib in pediatric patients with newly diagnosed or imatinib-resistant/-intolerant Ph+ CML in the chronic phase. The authors indicated that dasatinib is a first- and second-line option available for the treatment of Ph+ CML-CP and is well tolerated in pediatric patients [19].

In our study, one child with T315I and M244V mutation received ponatinib, which was started after the HR-1 course. We observed serious toxic events during ponatinib therapy, such as gastrointestinal disorders, hepatotoxicity, and skin inflammation. This patient had MUD-SCT, and the follow-up is six years. Millot et al. described the tolerance and effectiveness of the use of ponatinib in pediatric patients with Ph+ leukemias (11 children with CML and 3 with ALL). Ponatinib was used as a second- to eighth-line treatment. The T315I mutation was identified in four patients with CML and in one patient with ALL Ph+ in bone marrow relapse. Three children achieved molecular remission, one did not respond, and one relapsed. Hematologic toxicity grade III or IV occurred in two patients, and grade I or II occurred in three patients [20].

To conclude, our data show that the adverse events of imatinib therapy were not severe and were mostly grade I–II. In our opinion, the use of PCR-MRD to assess response to treatment would allow a more precise classification of patients into risk groups, and thus improve outcomes in Poland. This study significantly complements further research into TKIs in conjunction with a chemotherapy regimen for the treatment of pediatric patients with Ph+ ALL. This is essential because of the general outcome of children with ALL Ph+ remains unsatisfactory.

## 4. Materials and Methods

### 4.1. Study Group

The parents or guardians of patients under the age of 18 years gave permission for their child to be enrolled in the study. All procedures performed in studies involving human participants were in accordance with the ethical standards of the institutional and/or national research committees (the Ethics Committee of the Medical University of Lublin. Ethical code: KE-0254/222/2012) and with the 1964 Helsinki Declaration and its later amendments, or comparable ethical standards.

In this study, we analyzed the medical records of 31 patients, aged 1–18 years, with newly diagnosed ALL Ph+, who were treated according to the EsPhALL2010 protocol in 15 hemato-oncological centers from 2012 to 2019 in Poland. Patient clinical data were collected at diagnosis, along with data of treatment response on day 8 (response to steroids) and on days 15 and 33 of therapy (results of bone marrow). Day 15 MRD measurement by flow cytometry was available for our patients.

Conventional cytogenetics (CC) and FISH were used for the identification of genetic changes in BM at diagnosis in each patient according to the standard protocols. Chromosomes were prepared from a 24 h unstimulated reference culture. Conventional cytogenetic analysis has limitations, which result from cell culture failure, low mitotic index, or poor quality of the morphology of metaphase chromosomes. The limitations of banding cytogenetic analysis related to bone marrow cell culture failure were considered. The molecular karyotype obtained by using microarrays enables the detection of missing or hidden aberrations among cases with failed or normal cytogenetics, but this protocol of treatment was not available for our patients.

FISH was performed in patients using commercially available probes (Vysis LSI BCR/ABL Dual Color, Dual Fusion Translocation Probe Abbott Molecular, Des Plaines, IL, USA) in accordance with the manufacturer’s instructions. The median percentage of BCR/ABL1 cells in our patients was 69.8% (detected by the FISH method).

### 4.2. Treatment and Supportive Care

According to the protocol, patients were stratified into risk groups—good or poor—depending on prednisone response (blast cell count of ≤1000/µL in peripheral blood after seven days of prednisone and a single intrathecal dose of methotrexate), ≤25% marrow blasts at day 15, or <5% marrow blasts at day 21, depending on national induction protocols (at day 33 in Poland).

**Scheme 1:**
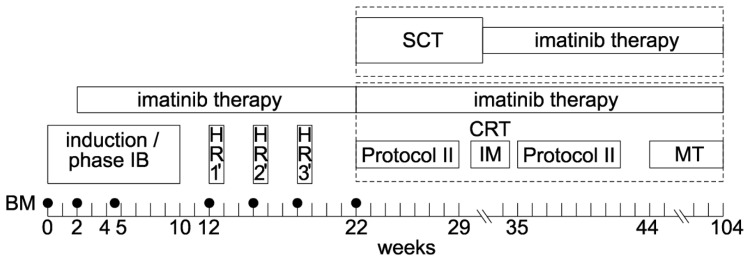
Characteristics of the therapeutic protocol. BM: bone marrow; SCT: stem cell transplantation; HR: high risk; IM: interim maintenance; MT: maintenance therapy; CRT: cranial radiotherapy.

The treatment with the EsPhALL2010 protocol included the following phases: induction (according to the national/study group treatment protocol), consolidation (HR blocks), reinduction (two protocol II courses) with interim maintenance (cranial radiation during this phase for all patients who were not transplanted), and maintenance therapy (Scheme 1). The induction phase for the Polish children included prednisone, vincristine (four doses), daunorubicin (four doses), and L-asparaginase (12 doses). Imatinib at a dose of 300 mg/m^2^ daily was introduced on day 15 of induction in all patients. All children were also screened for an HLA (human leukocyte antigens) identical family member or unrelated donor. HSCT was determined after the third HR block. Imatinib was administered throughout the first year post-transplantation until day +365 of HSCT (dose 200–300 mg/m^2^ daily depending on tolerance). The toxic events were evaluated according to the National Cancer Institute (NCI) Common Toxicity Criteria, version 2.0. Supportive care and therapy modifications of imatinib toxicity were used as recommended by the protocol. Good-risk patients were classified for HSCT when there was a genotype-matched donor (9/10 or 10/10), and high-risk ones were classified as such with any type of donor (matched or mismatched family donor, unrelated or haploidentical donor) [6].

### 4.3. Statistical Analysis

Nominal variables are presented as *n* (% of group), while continuous variables are presented as mean (SD) or median (range), depending on the normality of their distribution. Data normality was verified via Shapiro–Wilk test and based on visual assessment of histograms. Nominal variables in total group were analyzed with chi-square goodness-of-fit test to verify if observed frequencies are significantly different from the expected frequencies. OS and EFS curves were prepared with the use of the Kaplan–Meier survival analysis method, including 95% confidence intervals. The log-rank Cox–Mantel test was used to compare OS and EFS levels between subgroups. The cumulative death and relapse risk curves were estimated based on the Kaplan–Meier method with 95% confidence intervals. All tests were two-tailed at the level of significance α = 0.05. Analysis was conducted in R software, version 3.5.4.

## 5. Conclusions

This study is valuable because it was based on the clinical data of patients in a rare subgroup of children with ALL Ph+ who were treated according to a personalized treatment protocol in several pediatric centers in Poland. Our treatment outcomes are still disappointing compared to those in other reports. Improvements in supportive care and emphasis placed on the determination of MRD at successive time points, which will impact decisions on therapy, may be required. Comparing with other studies, we observed a real need for an additional method of detecting MRD using a PCR method in Poland. We observed that imatinib was well-tolerated, with no serious complications.

## Figures and Tables

**Table 1:** Clinical characteristics of children with ALL Ph+ (Philadelphia chromosome positive acute lymphoblastic leukemia).

Patients’ Features	*n* = 31 (%)	*p*-Value ^1^
Sex		
Female	13 (42)	
Male	18 (58)	0.37
Median age (years)	9.4	
CNS infiltration		
Yes	4 (12.9)	
No	27 (87.1)	<0.001
Organ infiltration		
Testes	2 (6.5)	<0.001
Liver	19 (61.3)	0.2
Mediastinum	0 (0)	n/a
Lymph nodes	8 (25.8)	0.01
Spleen	15 (48.4)	0.86
Risk group		
Good	9 (29)	
Poor	21 (67.7)	0.03
Immunophenotype		
Pre-B	29 (93.5)	
T-ALL	2 (6.5)	<0.001
WBC at diagnosis (µL)		
<20,000	9 (29)	
≥20,000	22 (71)	0.02
Genetic aberration ^2^		
Karyotype with *BCR/ABL1* aberration	22 (71)	0.02
Karyotype with *KMT2A* rearrangement	0 (0)	n/a
*KMT2A/AFF1 (formerly MLL-AF4)*	0 (0)	n/a
*ETV6/RUNX1*	0 (0)	n/a
Hypodiploidy	2 (6.5)	<0.001
Hyperdiploidy	3 (9.7)	<0.001
Chromosome 7 aberration (*IKZF1*)	4 (12.9)	<0.001
Complex karyotype	10 (32.3)	0.05
Prednisone response		
Good	17 (54.8)	
Poor	14 (45.2)	0.59
% blast (BM) day 15		
M1	15 (48.4)	
M2	4 (12.9)	
M3	12 (38.7)	0.04
FCM-MRD (%) day 15		
<0.1	2 (6.5)	
≥0.1 and <10	12 (38.7)	
≥10	17 (54.8)	0.004
% blast (BM) day 33		
M1	29 (93.5)	
M2	1 (3.2)	
M3	1 (3.2)	<0.001

^1^ analysis with chi-square goodness of fit test; ^2^ It is recommended that gene and allele symbols are underlined in manuscript and italicized in print; CNS: central nervous system; WBC: white blood cell; BM: bone marrow; FCM-MRD: flow cytometry minimal residual disease; M1: blast < 5%, M2: blast ≥ 5% < 25%, M3: blast ≥ 25%.

**Table 2:** The grade of the toxicity for each patient. TKIs: tyrosine kinase inhibitors.

TKIs	Toxicity																								
	Grade I						Grade II						Grade III						Grade IV						
	Infection	Hepatotoxicity	Gastrotoxicity	Dermatitis	Neurotoxicity	Nephrotoxicity	Infection	Hepatotoxicity	Gastrotoxicity	Dermatitis	Neurotoxicity	Nephrotoxicity	Infection	Hepatotoxicity	Gastrotoxicity	Dermatitis	Neurotoxicity	Nephrotoxicity	Infection	Hepatotoxicity	Gastrotoxicity	Dermatitis	Neurotoxicity	Nephrotoxicity	Patient
Imatinib																									1
																									2
		x					x	x		x															3
							x		x	x															4
							x	x	x																5
							x				x														6
								x	x																7
		x					x				x														8
							x																		9
																									10
																									11
																									12
																									13
										x															14
																									15
																									16
								x				x													17
																									18
							x	x			x	x													19
														x											20
							x	x	x																21
												x													22
																									23
																									24
										x			x		x										25
							x	x	x																26
																									27
Dasatinib							x						x												28
							x	x	x		x														29
							x	x	x	x															30
Ponatinib														x	x							x			31
